# Yellow Fever

**Published:** 1888-09

**Authors:** 


### YELLOW FEVER.

Yellow fever has again appeared in the South, and the authorities at Jacksonville, Florida, are battling with it without any satisfactory result thus far. The lateness of the season when it appeared and the exceptionally cool weather of this summer give hope that, with the efficient quarantine regulations that it is said are being enforced, the disease may be confined to the territory now infected, and that it may soon be under control. The occasion affords an opportunity of testing the method of treatment suggested by Surgeon Sternberg of the United States Army, as the result of his recent investigations in the West Indies.

